# Tandem repeats modify the structure of human genes hosted in segmental duplications

**DOI:** 10.1186/gb-2009-10-12-r137

**Published:** 2009-12-02

**Authors:** Anna De Grassi, Francesca D Ciccarelli

**Affiliations:** 1 Department of Experimental Oncology, European Institute of Oncology, IFOM-IEO Campus, Via Adamello, 20139 Milan, Italy

## Abstract

**Background:**

Recently duplicated genes are often subject to genomic rearrangements that can lead to the development of novel gene structures. Here we specifically investigated the effect of variations in internal tandem repeats (ITRs) on the gene structure of human paralogs located in segmental duplications.

**Results:**

We found that around 7% of the primate-specific genes located within duplicated regions of the genome contain variable tandem repeats. These genes are members of large groups of recently duplicated paralogs that are often polymorphic in the human population. Half of the identified ITRs occur within coding exons and may be either kept or spliced out from the mature transcript. When ITRs reside within exons, they encode variable amino acid repeats. When located at exon-intron boundaries, ITRs can generate alternative splicing patterns through the formation of novel introns.

**Conclusions:**

Our study shows that variation in the number of ITRs impacts on recently duplicated genes by modifying their coding sequence, splicing pattern, and tissue expression. The resulting effect is the production of a variety of primate-specific proteins, which mostly differ in number and sequence of amino acid repeats.

## Abstract 2

Internal tandem repeats are shown to modify the gene structure of human primate-specific paralogs.

## Background

The completion of the human genome and recent advances in sequencing technologies have revealed the presence of recently duplicated genomic segments with high degrees of sequence identity. Some of these regions have reached fixation during primate evolution and are known as segmental duplications (SDs) [1]. Other segments are still polymorphic and represent copy number variants (CNVs) within the human population [2-4]. Duplicated blocks are usually enriched in genes, thus providing raw material for the evolution of novel gene families [5-7].

Newly duplicated paralogs undergo rearrangements that usually cause their non-functionalization [8]. Sporadically, these modifications lead to advantageous events, such as the development of a novel function (neo-functionalization) or the repartition of the original function between paralogs (sub-functionalization). Under these circumstances, the new genes are rapidly preserved and fixed into the population [9-14]. Rapid divergence of paralogs immediately after gene duplication is a consequence of the relaxed evolutionary pressure that favors the retention and the propagation of the mutated alleles [8,15]. In this particular context, errors in DNA replication act as a major source of evolutionary innovation.

One of the most frequent replication errors involves internal tandem repeats (ITRs), which are short genomic regions that undergo homologous unequal crossing-over and replication slippage. ITRs are very frequent in eukaryotic genomes [16] and show a positive correlation with genome size in metazoans [17,18]. The biological role of ITRs has been a matter of long-standing debate. In a gene-centric view of genome evolution, these regions have been often tagged as junk DNA, particularly when they localize in intergenic segments and within introns. However, growing evidence has shown that ITRs are important for the evolution of eukaryotic genomes because they act as potential source of genetic variation owing to their 'mutator properties' [19]. Several examples supporting this role have been accumulating over the years, including cell adhesion in yeast [20], morphological modifications in dogs [21], social behaviors in voles [22], and differences in sexual behavior between primates [23]. In coding exons, repetitions mostly involve trinucleotides due to selection against frameshift [24]. In this context, ITRs, and particularly short repeats or microsatellites (< 10 bp), are highly polymorphic within the human population. Polymorphic trinucleotides are often associated with human genetic diseases, one of the best-known examples being the expansion of polyglutamine traits in Huntington disease and various other spinocerebellar ataxias (for recent reviews, see [25,26]). The association between trinucleotide polymorphisms and genetic diseases might lead to the conclusion that repeat variations are always evolutionarily deleterious. However, this is not true: the CAG repeat of the *SCA2 *gene is under positive selection within the CEU population, although the biological reasons for this selection are still unknown [27].

We have recently shown other possible evolutionary outcomes deriving from ITR variations. Namely, we described the structural modifications occurring in *PRDM7*, a primate-specific member of the *PRDM *gene family, where the repeat contributes to the acquisition of a complex pattern of splicing variants and tissue-specific expression [14]. In the present study, we extend the analysis to all variable ITRs in human paralogs lying in SDs with the aim of verifying whether ITR-driven modifications represent a widespread mechanism for the evolution of novel genes.

## Results

### ITRs modify around 7% of human genes hosted in segmental duplications

**Figure 1:**
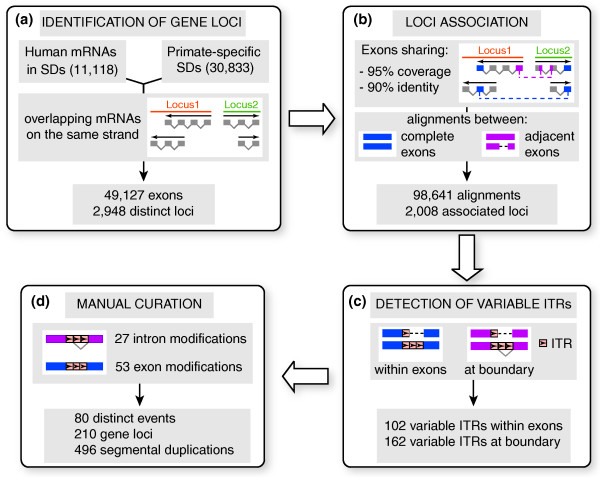
Genome-wide detection of ITR-driven gene modifications. **(a) **The set of human gene loci within human SDs was retrieved. Each locus is composed of transcripts that overlap on the same strand. **(b) **After an all-against-all alignment between exons, only loci that share at least one exon with 95% coverage and 90% sequence identity were kept. The alignments could involve complete exons (blue) or portions of adjacent exons (pink). **(c) **From this dataset, alignments with a variable number of ITR units were extracted. **(d) **The effect of the variable ITR on the gene structure was manually checked to remove false positives and discriminate between exon and intron modifications.

SDs are regions of the human genome longer than 1 kb, with at least 90% sequence identity, and that underwent duplications during the last 25 million years of primate evolution [28]. We grouped all human genes hosted in SDs into 2,948 discrete gene loci, each composed of all overlapping mRNAs lying on the same DNA strand (Figure 1a). This definition of gene loci allowed the identification of human genes hosted within SDs by using directly the genome annotation rather than pre-compiled collections of genes. We performed an all-against-all alignment between all exons in these gene loci to extract the 2,008 loci that are associated through nearly identical exons (Figure 1b). We only considered for further analysis the exon alignments with a variable number of ITRs between the aligned exons (Figure 1c). By looking at the exon-intron structure of the corresponding genes, we identified 102 alignments with variable ITRs retained within both exons and 162 alignments with ITRs occurring at exon-intron boundaries (Figure 1c). After manual inspection to eliminate false positives (see Materials and methods), we identified 53 exon and 27 intron modifications (Figure 1d). We carefully analyzed the transcription evidence supporting each of these modifications to exclude that they were artifacts of RNAs with multiple matches on highly identical genomic sequences, such as SDs. Strikingly, almost all loci with variable ITRs (> 96%) are supported by RNAs with unique or best matches, and only less than 4% are associated with ambiguous transcription evidence (Table 1). This result confirms that ITR-driven gene modifications are real events and not artifacts of genome mapping.

**Table 1:** Gene loci, segmental duplications and transcription evidence associated with internal tandem repeat-driven modifications

			RNA support		
			
Modifications	Associated gene loci	Associated SDs	Unique match	Best match	Multiple match
Exon (53)	180	474	48	5	2
Intron (27)	106	370	21	4	1
Both (18)	76	154	-	-	-
Total (80)	210	496	68	9	3

In 18 cases, the same variable ITR can result in both exon and intron modifications. RNAs supporting the modification can have a unique or best match in that locus, or multiple matches in the genome (see Materials and methods).

Due to the multiple rounds of duplications during primate evolution, the same modification could be found in several gene loci. Overall, the 80 modifications were detectable in 210 gene loci, lying within 496 SDs (Table 1; Additional file 1). Variable ITRs therefore affect 7% (210 out of 2,498) of the human genes hosted in primate-specific duplications.

### Variable ITRs occur in large groups of recently duplicated paralogs

**Figure 2:**
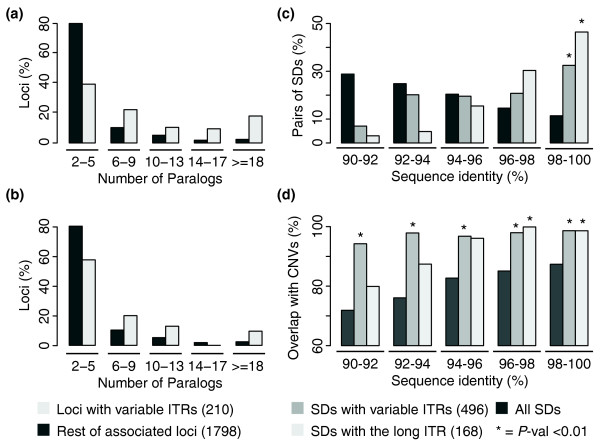
Number of paralogs and age of the loci with variable ITRs. **(a) **Comparison between the number of paralogs of the 210 loci with variable ITRs and the remaining 1,798 nearly identical loci. The former are enriched in large groups of paralogs. **(b) **The same trend is observed when only the paralogs directly hosting ITR-containing exons are compared to the rest. **(c) **Sequence identity between all pairs of SDs (25,914), pairs of SDs with variable ITRs (496), and pairs of SDs with the longest version of each ITR (168). The last two are enriched in highly identical SDs. **(d) **Overlap between SDs and human CNVs. Both SDs with variable ITRs and SDs with longer versions of each ITR tend to overlap with human CNVs. *P-value < 0.01 (chi-squared test between the corresponding fraction of SDs and all SDs in that bin of sequence identity).

To better characterize the genes that undergo ITR-driven modifications, we compared the paralogs of the 210 loci with variable ITRs with those of the remaining 1,798 nearly identical loci. We counted the number of paralogs of the 210 loci that were associated through any nearly identical exon, and through only ITR-containing exons. Both comparisons showed that the 210 loci with variable ITRs are significantly enriched in larger groups of paralogs (*P*-value < 10^-3^, Wilcoxon test; Figure 2a, b). The same trend is detectable also when exon and intron modifications are analyzed separately (*P*-value < 10^-3^; Figure S1 in Additional file 2). These data suggest that ITR-driven modifications have occured in genes that underwent several rounds of duplications. This is not surprising as these genes had higher chances to undergo modifications and likely experienced periods of relaxed evolutionary pressure due to functional redundancy [8].

Interestingly, SDs with variable ITRs tend to occur within the same chomosome more frequently than all other SDs (70.4% and 39.3%, respectively, *P*-value < 10^-3^, chi-squared test). Since intrachromosomal SDs are known to be recent duplications [29], this enrichment may suggest that ITR-driven gene modifications occurred recently during primate evolution. To be able to date the appearance of the loci with variable ITRs during primate evolution, we relied on the percentage of identity between pairs of SDs, which returns an indication of when the duplication occurred in time [30,31]. When compared to the rest, the 496 SDs hosting the loci with variable ITRs are enriched in recent SDs (Figure 2c). In particular, 161 of them (32.5%) share more than 98% sequence identity and hence underwent duplication during or after the speciation between human and chimpanzee [30]. This percentage is significantly higher compared to all SDs (11.4%) and increases to 46.4% when, for each of the 80 modifications, only the 168 SDs with the longest version of the repeat are considered (*P*-value < 10^-3^, chi-squared test; Figure 2c). Genes with variable ITRs, and especially those with the longest ITR version, have formed through recent duplications.

### Genes with variable ITRs lie in polymorphic regions of the human genome

The results reported above may suggest that the 496 SDs bearing variable ITRs continue to undergo further rearrangements and fixation in the human population. We therefore measured the co-occurrence of SDs with variable ITRs and human CNVs, which are large polymorphic regions (> 1 kb) of the human genome accounting for a large portion of human variation [3,32]. We observed the expected general trend [30,33,34] in which recent SDs tend to undergo variation within the human population (Figure 2d). However, when only SDs with variable ITRs are considered, they are significantly more represented within human CNVs, independent of the age of the SD. Also in this case, the signal is still detectable when only SDs with the longest version of the repeat are considered (Figure 2d). This observation suggests that ITR-driven modifications preferentially occur in evolutionarily dynamic regions of the genome that are still undergoing modification within the human population.

The fact that SDs with the longest version of the ITRs are particularly enriched in recent SDs as well as in human CNVs may indicate that the direction of ITR modifications within the primate lineage is towards expansion more than contraction, possibly through replication slippage or unequal crossover. To further verify this, we counted the number of ITRs in the orthologous exons of two outgroup species, mouse and dog. For 11 out of 80 modifications we could detect no orthologous sequence (Additional file 1), suggesting that the exon itself originated in primates. For the remaining 69 ITR modifications, at least one ortholog was recovered in mouse or dog. In all cases but two (*ZNF100 *and *FOXD4L*) the number of ITRs was higher in human than in the other species. This result confirms that variable ITRs in SDs mostly expanded in the primate lineage, resulting in exon and intron elongations.

### ITR-driven modifications are due to expansion of minisatellites

**Figure 3:**
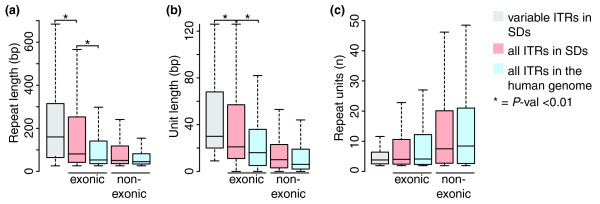
Length of variable ITRs compared to all ITRs in SDs and in the human genome. Compared are **(a) **the total length of the repeats, **(b) **the length of the repeat unit, and **(c) **the number of repeat units between the variable ITRs that modify the gene structure (grey) and all other exonic and non-exonic ITRs in SDs (pink) and in the rest of the human genome (light-blue). ITR modifications occur preferentially through the repetition of minisatellites and are depleted in short repeats.

Variable ITRs responsible for gene modifications are composed, on average, of 30-bp units that are repeated 4 times for a total length of 160 bp (Table S1 in Additional file 2). When compared to all ITRs within exonic and non-exonic regions hosted in SDs as well as in the whole human genome, variable ITRs affecting the gene structure are significantly longer (Figure 3a) as a consequence of longer units (Figure 3b) rather then of higher numbers of repetitions (Figure 3c). Therefore, ITR-driven modifications of genes hosted in SDs are preferentially mediated by minisatellites. This result can be explained by different and concomitant reasons. First, it partly reflects the fact that we focused on almost identical regions, thus favoring the detection of longer repeat units. As a general trend, ITRs lying in SDs have, on average, repeat units significantly longer than ITRs dispersed in the rest of human genome (Figure 3b). Second, long repeats are more variable than short repeats probably because they enlarge the target sequence for slippage or unequal crossover [35]. Finally, the absence of variable ITRs with repeat units shorter than 9 bp (Figure 3b) suggests a preferential retention of repeats that can significantly diversify the sequence of the encoded proteins.

### Fifty percent of variable ITRs modify protein sequences

In agreement with an active role in modifying protein sequences, we found that 50% of the detected ITRs occur in the coding sequence of genes lying in SDs (Table 2). This is different, for example, from smaller ITRs in housekeeping genes, which preferentially occur within untranslated regions [36]. We manually analyzed all these 40 modifications in order to verify the effect of the repeats on the resulting proteins. In the majority of cases, the reading frame of the original protein is preserved and variable ITRs cause the elongation of low complexity regions in between globular domains as well as of amino acid repeats, such as zinc fingers and protein-specific repeats (Table S2 in Additional file 2). Often these modifications occur in polymorphic human proteins, such as the keratin-associated proteins, the VCX/Y proteins, the nuclear pore interacting proteins, and the prostate-ovary-testis-endometrium proteins. In this latter case, the formation of an amino acid repeat is involved in the modification of the protein's cellular localization [37].

**Table 2:** Occurrence of variable internal tandem repeats within coding and non-coding transcripts

			Coding sequences		
			
Modification	Non-coding RNAs	UTRs	ITR unit (bp)	In-frame	Out of frame
Exon (53)	10	13	< 40 (9-39)	14	3
			> 60 (63-228)	9	4
Intron (27)	9	8	< 40 (9-30)	3	0
			> 60 (62-126)	6	1

The number of ITR modifications localized in non-coding mRNAs, untranslated regions (UTRs), and coding sequences is reported. For ITRs lying within coding sequences, the length of the ITR unit in base pairs and the effect on the reading frame of the encoded proteins are also shown.

In seven cases variable ITRs introduce frame shifts with the formation of novel amino acid sequences (Table S2 in Additional file 2). Although no specific functional assignment has been made so far for any of these new sequences, they represent an innovation in terms of amino acid composition within the primate lineage. In all seven cases ITR modifications occur in the last coding exon, thus producing an accretion of the protein sequences without affecting the original composition. Moreover, this also suggests that the resulting mRNAs are potentially able to escape nonsense-mediated mRNA decay [38] and produce functional proteins.

### ITRs are involved in the diversification of *Morpheus *paralogs

**Figure 4:**
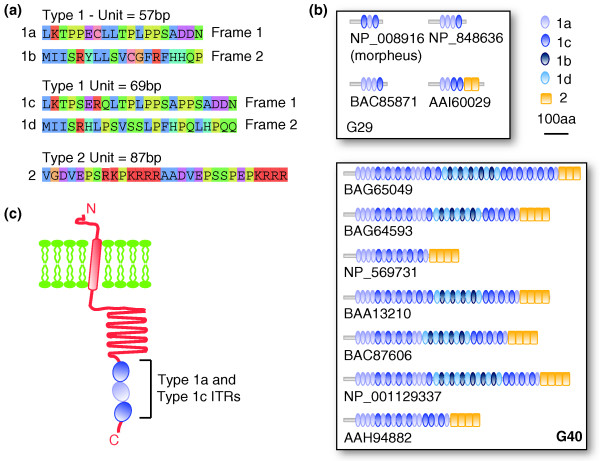
Effect of variable ITRs on the coding sequence of nuclear pore interacting proteins. **(a) **Amino acid sequences encoded by the ITR units of the human *morpheus *paralog BAG65049 were taken as representatives. Type 1 repeats are associated with two different units of 57 bp and 69 bp, respectively, and are translated into two different frames. This results in four distinct amino acid repeats (1a, 1b, 1c and 1d). Type 2 repeats are much simpler and only produce one sequence. Amino acids are highlighted according to the Clustal color scheme [71]. **(b) **Representation of the protein sequences encoded by the human paralogs of exon 8 of *morpheus*. Only proteins associated with transcripts shown in Table 3 are reported. **(c) **Cartoon of the possible three-dimensional structural organization of *morpheus*, based on secondary structure predictions (see text). These predictions were confirmed also for the other RefSeq transcripts. The representation is not to scale.

One of the most complex cases of ITR-driven modification that we identified involves the paralogs of *morpheus*, a primate-specific gene under strong positive selection in hominoids [11]. To investigate the role of variable ITRs in the diversification of potentially important genes in human evolution, we manually analyzed and reconstructed the structure of the ITR-containing exons in this family. Overall, we identified 26 paralogous loci, most of which have uniquely or best mapping transcripts that encode nuclear pore interacting proteins (NPIPs; Table 3). All these genes but one are hosted in a region of human chromosome 16 that underwent several rounds of duplications during primate evolution [39-41]. The paralogous exons can host two different ITRs, namely type 1 and type 2 repeats (Figure 4a). Type 1 repeats are associated with two different units of 57 bp and 69 bp, respectively, which in turn can be translated into two different frames. As a result, type 1 repeats can produce four distinct amino acid sequences. Type 2 ITRs are much simpler repeats with a single 87-bp unit and a unique reading frame. Depending on the percentage of identity between exons, *morpheus *paralogs can be assembled into two groups (G29 and G40; Additional file 1), which also reflect a different degree of expansion of the ITR units. Members of G29 host a maximum of four repeats of type 1 and two repeats of type 2, while members of G40 show a large repeat expansion, with up to 39 copies of type 1, and 4 repeats of type 2 (Table 3). Not all variable ITRs present at the genomic level are also found in the mature transcripts and often the same locus is associated with transcripts that differ in the number of ITRs (Figure 4b). However, rather than being due to a complex pattern of alternative splicing, this variability seems the result of the high structural polymorphism of these regions in the human population. All *morpheus *loci but two overlap with human CNVs, and in at least three cases the polymorphic region corresponds to the repeats (Table 3). Interestingly, there are only two paralogs with less than three ITRs of type 1 and in none are the ITRs in coding exons, suggesting that at least three units of type 1 repeat are required for protein function.

**Table 3:** Features of variable internal tandem repeats present in paralogs of the human *morpheus *gene

			ITRs in genome					ITRs in mRNA	
							
Group ID	Genomic locus of exon 8	Exon length (bp)	Type1	Type2	CNVs	Associated proteins or transcripts	Exon length (aa)	Type1	Type2

G29	chr.16:68567795-68568136	342	1	0	1	NR_003610, PDXC2 * (U)	-	1	0
	chr.18:11928734-11929237	504	2	2	0	-	-	-	-
	chr.16:14952973-	408	3	0	4	BC023572 * (M)	-	2	0
	14953380					NP_008916, morpheus (B)	136	3	0
	chr.16:15365020-15365427	408	3	0	2	-	-	-	-
	chr.16:11609511-11609918	408	3	1	0	-	-	-	-
	chr.16:28261432-28262010	579	3	2	1	-	-	-	-
	chr.16:15105678-15106116	439	4	0	1	-	-	-	-
	chr.16:16351450-16351914	465	4	0	3	BAC85871 (U)	155	4	0
	chr.16:16394814-16395312	465	4	0	3	NP_848636 (M)	155	4	0
	chr.16:18319329-18319793	465	4	0	5	NP_848636 (M)	155	4	0
	chr.16:18359482-18359946	465	4	0	5	NP_848636 (M)	155	4	0
	chr.16:28375249-28375848	600	4	2	2	-	-	-	-
	chr.16:28690975-28691574	600	4	2	4	-	-	-	-
	chr.16:28576850-28577449	600	4	2	5	-	-	-	-
	chr.16:72982790-72983476	687	4	2	5	AAI60029, NPIPL2 (U)	229	4	2
	chr.16:28970894-28971553	660	4	2	1	-	-	-	-

G40	chr.16:22452448-22455204	2757	35	4	12	NP_001129337 (U)	919	35	4
						AAH94882 (B)	544	18	4
						NP_569731, NPIPL3 (B)	464	14	4
	chr.16:29300271-29303111	2841	37	3	2^†‡^	-	-	-	-
	chr.16:30141785-30144625	2841	37	4	2	BAC87606 (B)	794	29	4
	chr.16:21321092-21324115	3024	39	4	5^†§^	BAG65049 (U)	970	39	3
						BAG64593 (U)	849	31	4
						NP_569731 (B)	464	14	4
	chr.16:21753543-21756566	3024	39	4	10^†^	BAA13210 (B)	840	31	4

The paralogous sequences of *morpheus *exon 8 were detected and characterized for the presence and number of type 1 and 2 ITRs. Group IDs refer to Additional file 1. Exon length is reported in base pairs and in amino acids (aa) only when supported by coding mRNAs. Unique (U), best (B) or multiple (M) transcript support is reported for each locus. CNVs that overlap the genomic locus of exon 8 are indicated. *ITRs residing in non-coding RNAs. ^†^CNVs overlapping exclusively with ITRs, and corresponding to identifiers ^‡^30774, ^§^30757 and 30761 of the Database of Genomic Variants.

The reason for the high variability in the number of ITRs in the morpheus family is currently unknown. Together with positive selection occurring at the upstream exons 2 and 4 of *morpheus *[11], it could be a sign of the fixation process that the entire family is currently undergoing in hominoids. Interestingly, the protein encoded by *morpheus *localizes at the nuclear membrane, where it interacts with the nuclear pore complex [11]. According to several secondary structure predictors [42-46], *morpheus *hosts a transmembrane segment in its amino-terminal part, followed by a helical portion before the repeats (Figure 4c). Variation in amino acid repeats is a known mechanism to vary the surface of interaction with different targets [47]. This could be the case also for the different paralogs of *morpheus*, which in this way could adapt and fine-tune their binding to different interactors.

## Discussion

Tandem repetitions of short sequences represent an effective case of 'dynamic mutations' in which a secondary event can easily occur after the primary duplication [48]. As a consequence of such high dynamism, it is not surprising that ITRs highly differ between lineages [49,50], species [51], and even individuals [52]. In this study we show that long and variable ITRs are involved in the modification of 7% of human genes hosted in primate-specific SDs. These genes are very recent and therefore likely to be still in the process of formation and fixation. Confirming their evolutionary dynamism, genes with variable ITRs are enriched in human variant regions, in spite of the overall paucity of CNVs that overlap with RefSeq genes [53]. At least 50% of variable ITRs contribute to the modification of coding sequences, mostly leading to the elongation of amino acid repeats in the encoded proteins. When variable ITRs occur at the exon-intron boundary, they may cause the formation of novel introns (Table S3 in Additional file 2). The majority of such intron modifications (66% of the total; Table 1) also show support for the alternative transcript, in which the repeat is retained within the exon. Alternative transcripts occur less frequently in exon modifications (34%, *P*-value = 0.01, chi-squared test), suggesting that the formation of novel introns is a more complex event that requires further rearrangements to generate novel splice sites [54]. For some of the reported cases, variable ITRs cause the activation of cryptic splice sites and the formation of novel introns [55]. This model of intron formation has so far been invoked only very seldom [14,56,57], likely because the fast divergence of intronic sequences makes the identification of intron gains very challenging and often questionable [58-61]. Because our analysis focuses on recent events, it enables the capture of signs of intron formation before they disappear as a result of sequence divergence. None of the putative intron gains reported in our study had been previously identified, likely because our approach does not limit the search for repeated regions to intron boundaries [62] but extends it to the entire ITRs contained within exons. This approach also reduces the chance of false positives due to RT-PCR artifacts [63]. When no canonical splice sites can be identified (Table S3 in Additional file 2), the discrepancy between the number of putative ITRs between genomic and transcribed sequences can be better explained by structural polymorphisms more than by intron gains. This is the case of *morpheus *paralogs, where both ITRs associated with these genes undergo copy number variation within the human population (Table 3).

**Figure 5:**
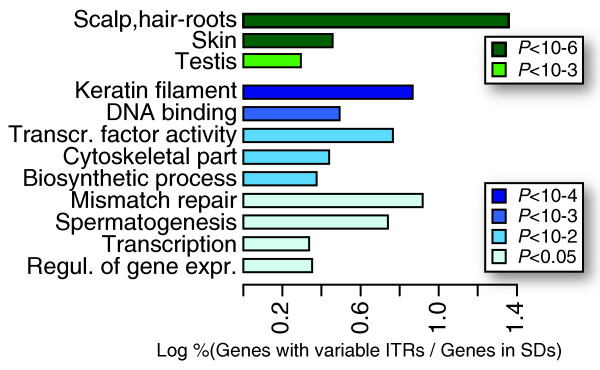
Functional enrichment of genes with variable ITRs. Tissue specificity (green) and functional enrichment (blue) of genes with variable ITRs compared to all other genes in SDs. The color gradient reflects the *P*-value of the chi-squared analysis.

While microsatellites can be involved in CNV formation [31,53], we found that the repetition of minisatellites seems to play a role especially in the diversification of recently acquired paralogs. How do these modifications affect the function of these genes? The general paucity of functional information on primate-specific genes prevents us from fully addressing this issue. Some hints can be derived, however, from the functional enrichment and the tissue expression of genes with variable ITRs. As expected for recent paralogs, genes with variable ITRs are significantly more expressed in skin and testis. Accordingly, the encoded proteins bind keratin filaments and are involved in spermatogenesis (Figure 5; Additional file 3). In agreement with previous reports [35], other over-represented functional categories are DNA binding, regulation of transcription and mismatch repair (Figure 5; Additional file 3). In these cases, the variable ITRs are preferentially located within the untranslated regions and thus probably involved in the regulation of transcription (G11 and G12; Additional file 1). Furthermore, variable ITRs can influence the tissue expression [14] and localization of the encoded protein [37], thus confirming that their presence actively modifies the gene function.

## Conclusions

In this study we show that some variable ITRs underlie recent changes in the structure of coding sequences, as well as changes in exon-intron boundaries. These modifications could constitute a mechanism for the evolution of novel gene arrangements. They especially occur in large groups of recently duplicated genes, which are also polymorphic in the human population. These ITRs are biased towards expansion of long units that can modify sequence, tissue expression and splicing patterns of newly formed paralogs. The focus on very recent modifications allows the observation of events that are usually very hard to detect, such as the formation of novel introns through activation of cryptic splice sites, and protein sequence accretion through the repetitions of long units.

## Materials and methods

### Identification of gene loci and detection of ITR-driven modifications

The genomic coordinates of 51,809 alignments between 30,833 primate-specific SDs [28] were recovered from the assembly of the human genome (hg18, March 2006) at UCSC [64]. Starting from 11,118 GenBank human RNAs lying within SDs for at least 95% of their sequence (UCSC mRNA track, frozen at May 2007), 2,948 discrete gene loci were derived by merging all RNAs mapping in the same locus and on the same strand. All exons within SDs were aligned using all-against-all BLAT [65]. Only pairwise alignments with at least 90% identity and covering at least 95% of the shortest exon were retained. Alignments between isoforms of the same exon and/or between unrelated SDs were discarded. From the resulting set, alignments bearing a different number of ITRs were extracted. ITRs were recovered from the UCSC simpleRepeat annotation track generated using Tandem Repeat Finder (TRF) [66]. As reported in the UCSC website, TRF was run under the following parameters: 2,7,7 = weights for match, mismatch and indels used in the Smith-Waterman local alignment; .80, .10 = matching and indel probability; 50 = minimum alignment score; 2000 = maximum size of the repeat unit. Applied to the entire sequence of the human genome, TRF is able to detect ITRs with a total length of 20 to 100,000 bp. Alignments were divided into two groups, according to the position of the variable ITRs in respect to the gene structure. ITR variation could occur either within exons, resulting in exon modification, or at exon-intron boundaries, leading to intron modification. For each modification, representative alignments were manually checked to eliminate false positives due to errors of TRF and/or incorrect alignment between repeats. The 524 RNAs associated with ITR modifications were carefully analyzed to verify the robustness of the transcription support for the new gene structure arrangements. Transcripts were classified as: unequivocally associated with the locus, if their only genomic match in terms of sequence identity and coverage corresponded to the locus with variable ITR; best associated with the locus, if the best genomic match corresponded to the locus with variable ITR; or multiply associated if they had multiple best matches on the genome.

### Groups of paralogs, age of SDs and overlap with human CNVs

For each of the 2,008 gene loci, the number of paralogs was defined as the number of loci associated by at least one nearly identical exon (> 95% coverage, > 90% identity). The resulting distributions of paralogs between the 210 loci with variable ITRs and the remaining 1,798 loci were compared, using the non-parametric Wilcoxon test. The percentage of identity between pairs of SDs was recovered directly from UCSC. A non-redundant set of 25,914 pair-wise alignments between SDs was derived, 496 of which involve the 210 loci with variable ITRs. The genomic coordinates of 21,178 human CNVs grouped in 6,558 non-overlapping CNV loci were recovered from the Database of Genomic Variants [67,68] (version 7, March 2009).

### Orthology assignment, tissue expression and functional enrichment

Orthologous regions corresponding to the human ITR-containing exons were extracted from the pair-wise BlastZ alignments between human and mouse (mm9) and human and dog (canFam2) using Galaxy [69]. The human/dog alignments were screened only in case the alignment between human and mouse was not available. The portions of the alignments corresponding to variable ITRs were manually checked. For 377 out of the 524 mRNAs with variable ITRs and for the 5,256 out of 8,638 mRNAs in 2,008 loci it was possible to extract information on the tissue type directly from GenBank. Tissues that were represented by at least 15 mRNAs with variable ITRs (> 3%) were selected for chi-squared comparison between transcripts with variable ITRs and other transcripts in SDs. Forty-nine percent of genes with variable ITRs can be associated with functional categories according to the Gene Ontology [70]. The functional enrichment was measured in comparison to other genes hosted in SDs. The functional terms in common between the two groups at levels 3 to 9 of the Gene Ontology hierarchy were compared using the chi-squared test and *P*-values were adjusted using the Bonferroni correction for multiple testing.

## Abbreviations

CNV: copy number variant; ITR: internal tandem repeat; SD: segmental duplication; TRF: Tandem Repeat Finder.

## Authors' contributions

FDC conceived and designed the study; ADG performed the experiments; ADG and FDC analyzed the data and wrote the paper.

## Additional files

The following additional data are available with the online version of this paper: an Excel file containing genomic coordinates and transcription evidence supporting ITR-driven gene modifications (Additional file 1); a Word file containing properties of variable ITRs, their effect on coding sequences and groups of paralogs associated with exon and intron modifications (Additional file 2); an Excel file providing functional analysis of genes with variable ITRs (Additional file 3).

## Supplementary Material

Additional file 1 For each group of paralogs, the genomic coordinates of exons, loci and repeats are reported, together with the transcriptional support. Click here for file

Additional file 2 Table S1: features of variable ITRs associated with modifications of the gene structure. Table S2: effect of variable ITRs on coding sequences. Table S3: effect of variable ITRs on introns. Figure S1: number of paralogs associated with intron and exon modifications. Click here for file

Additional file 3 For each over-represented category of the three main Gene Ontology classes (biological process, molecular function, and cellular component), the number and percentage of genes with variable ITRs and other genes in SDs are reported. Click here for file
