# Supplementary material for: Tandem repeats modify the structure of human genes hosted in segmental duplications
Source: Genome Biol. 2009 Dec 2;10(12):R137. doi: 10.1186/gb-2009-10-12-r137 (PMC2812944; doi:10.1186/gb-2009-10-12-r137)
Supplement: Additional file 2 — Table S1: features of variable ITRs associated with modifications of the gene structure. Table S2: effect of variable ITRs on coding sequences. Table S3: effect of variable ITRs on introns. Figure S1: number of paralogs associated with intron and exon modifications. [file gb-2009-10-12-r137-S2.DOC]

**Table S1. Features of variable ITRs associated with modifications of the gene structure.**

|  | Median | Minimum | Maximum |
| --- | --- | --- | --- |
|
| Total length | 160 | 20 | 1,869 |
| Unit length (bp) | 30 | 9 | 252 |
| Number of repeated units | 3.8 | 2 | 25 |

The median, minimum, and maximum values for each feature are reported (see also Additional Data File 1).

**Table S2. Effects of exon modifications on coding sequences.**

| **Frame** | **Parent Gene** | **Child Gene** | **ITR**  **Modification** | **ITR Unit** (bp) | **ITR Number** | **Effect on the Protein** | **GroupID** |
| --- | --- | --- | --- | --- | --- | --- | --- |
| Conserved | *POM121-2* | *POM121* | Formation | ITR1= 39  ITR2= 15 | ITR1= 2 ITR2= 3 | Low Complexity Region | G12 |
|  | *TSPY2* | *AK093413* | Formation | 9 | 5 |  | G18 |
|  | *FOXD4* | *FOXD4L* | Contraction | 11 | 2 |  | G36 |
|  | *NBPF3* | *NBPF10* | Formation | 14 | 4.5 |  | G27 |
|  | *BX647610** | *ZNF100* | Contraction | 84 | 12 | C2H2 zinc finger | G24 |
|  | *ZNF765* | *ZNF761* | Elongation | 84 | 18 |  | G25 |
|  | *ZNF37B** | *ZNF37A* | Formation | 252 | 2 |  | G39 |
|  | *KRTAP5-11* | *KRTAP5-2* | Elongation | 30 | 7 | KRTAP-specific repeat | G7 |
|  | *KRTAP4-8* | *KRTAP4-11* | Elongation | 15 | 20.5 |  | G22 |
|  | *KRTAP5-8* | *KRTAP5-3* | Elongation | 30 | 8.7 |  | G26 |
|  | *KRTAP12-1* | *KRTAP12-2* | Elongation | 30 | 5.6 |  | G28 |
|  | *KRTAP9-3* | *KRTAP9-4* | Formation | 15 | 2.3 |  | G38 |
|  | *KRTAP5-11* | *KRTAP5-10* | Elongation | 21 | 6 |  | G7 |
|  | *VCY* | *VCX2* | Formation | 30 | 2 | VCX-specific repeat | G17 |
|  | *VCY* | *VCX* | Elongation | 30 | 10 |  |  |
|  | *VCY* | *VCX3A* | Elongation | 30 | 8 |  |  |
|  | *POTEB** | *POTEH* | NA | 111 | 4 | POTE-specific repeat | G3 |
|  | *PDXDC2** | *NPIPL2* | NA | 69 | 2.5 | NPIP-specific repeat | G29 |
|  | *PRB4* | *PRB2* | Elongation | 63 | 17 | Proline-rich repeat | G35 |
|  | *LRRC37B* | *LRRC37A3* | Formation | 165 | 2.3 | Leucine-rich repeat | G37 |
|  | *SPANXE* | *SPANXB* | Formation | 9 | 3 | Tripeptide Repetition (NEA) | G16 |
|  | *SPDYE3* | *SPDYE1* | Formation | 75 | 2.2 | Protein Elongation | G11 |
|  | *SPDYE1* | *SPDYE2* | Elongation | 75 | 3.6 | Protein Elongation | G11 |
| Modified | *PRDM9* | *PRDM7* | Formation | 89 | 2 | Novel Sequence | G8 |
|  | *KIAA0220* | *LOC100132247* | NA | 126 | 7 |  | G40 |
|  | *BC118602** | *AK127145* | Elongation | 34 | 4 |  | G33 |
|  | *BX648930** | *AF332228* | Elongation | 20 | 3 |  | G10 |
|  | *IFITM3* | *X02490* | Formation | 68 | 2 |  | G13 |
|  | *FLJ46321* | *FLJ43859* | Formation | 26 | 2 |  | G15 |
|  | *UBB* | *BC070367* | Elongation | 228 | 4 |  | G20 |

All 30 cases for which the variable ITR occurs in the coding sequence of the gene were manually analyzed to verify the effect on the encoded protein. In 23 cases, the original reading frame was conserved, while in 7 it was modified. The parent and child gene represent the genes hosting a lower and a higher number of ITRs, respectively. The number of ITR units (ITR number) refers to the child gene. Group ID is the identifier for the paralog group used in this study. *= the repeat is localized in non coding portions of the gene.

**Table S3. Effect of intron modifications on coding sequences.**

| **Frame** | **Parent Gene** | **Child Gene** | **ITR Modifications** | **ITR Unit** (bp) | **ITR Number** | **Splice Sites** | **Group ID** |
| --- | --- | --- | --- | --- | --- | --- | --- |
| Conserved | *KRTAP4-11* | *KRTAP4-12* | Elongation | 15 | 25.5 | Canonical | G22 |
|  | *VCY* | *VCX3B* | Elongation | 30 | 14 |  | G17 |
|  | *TSPY2* | *TSPY3* | Formation | 9 | 5 |  | G18 |
|  | *PRDM9* | *PRDM7* | Formation | 89 | 2 |  | G8 |
|  | *PRH2* | *PRH1* | Formation | 63 | 2.4 |  | G35 |
|  | *PRB4* | *PRB1* | Elongation | 63 | 13 |  | G35 |
|  | *KIAA0220* | *NPIPL3* | NA | 126 | 7 | Non Canonical | G40 |
|  | *POTEB* | *POTEH* | NA | 111 | 4 |  | G27 |
|  | *PDXDC2** | *AF132984* | NA | 126 | 2.5 |  | G29 |
| Modified | *AK293845* | *HERC2* | Formation+ Elongation | 62 | 6 | Canonical | G1 |
| **Frame** | **Parent Gene** | **Child Gene** | **ITR Modifications** | **ITR Unit** (bp) | **ITR Number** | **Splice Sites** | **Group ID** |
| Conserved | *KRTAP4-11* | *KRTAP4-12* | Elongation | 15 | 25.5 | Canonical | G22 |
|  | *VCY* | *VCX3B* | Elongation | 30 | 14 |  | G17 |
|  | *TSPY2* | *TSPY3* | Formation | 9 | 5 |  | G18 |
|  | *PRDM9* | *PRDM7* | Formation | 89 | 2 |  | G8 |
|  | *PRH2* | *PRH1* | Formation | 63 | 2.4 |  | G35 |
|  | *PRB4* | *PRB1* | Elongation | 63 | 13 |  | G35 |
|  | *KIAA0220* | *NPIPL3* | NA | 126 | 7 | Non Canonical | G40 |
|  | *POTEB* | *POTEH* | NA | 111 | 4 |  | G27 |
|  | *PDXDC2** | *AF132984* | NA | 126 | 2.5 |  | G29 |
| Modified | *AK293845* | *HERC2* | Formation+ Elongation | 62 | 6 | Canonical | G1 |

All 10 cases for which the ITR is associated with an intron in the coding sequence of the gene were manually analyzed to verify the effect on the encoded protein. In 9 cases, the original reading frame was conserved and one or more additional ITRs were spliced out, while only in one case the frame was modified. The parent and child genes represent the gene hosting a lower or higher number of ITRs, respectively. ITR number refers to the child gene. Group ID is the identifier of the paralog group used in this study. *= the repeat is localized in non coding portions of the gene.

**Figure S1. Number of paralogs associated with intron and exon modifications.**

**­
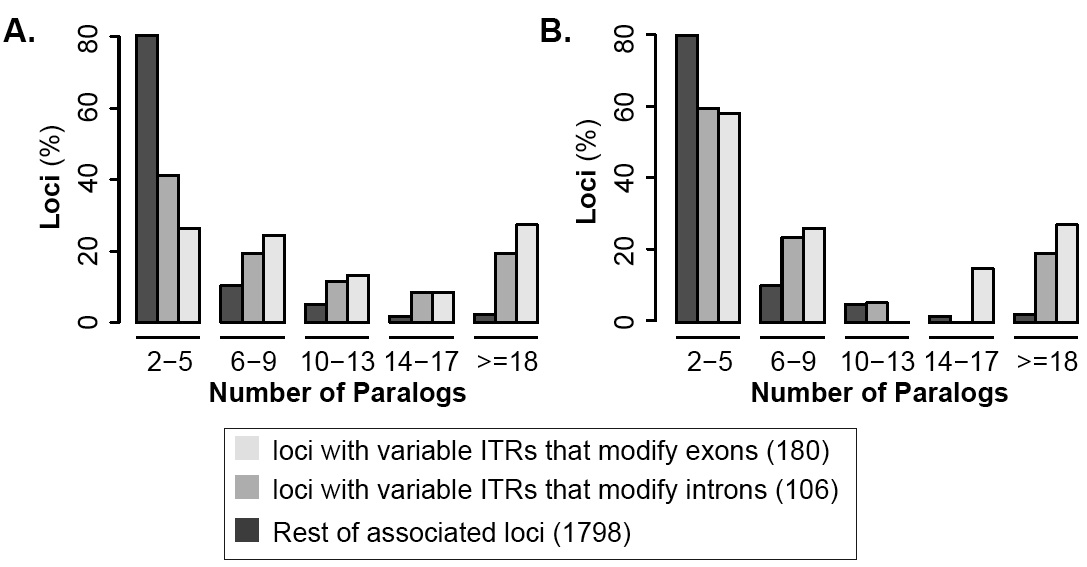
**

(A) Number of paralogs of 180 loci with exon modifications and 106 loci with intron modifications are compared to the remaining 1,798 loci. (B) Comparison after removing paralogs not directly associated by the repeat-containing exon. As seen for the entire dataset of 210 loci with variable ITRs (see main text), also these comparisons show that both exon modifications and intron modifications occur in large gene families.
